# Lymph node metastases outside tumor-bearing lobes and/or segments in non–small cell lung cancer

**DOI:** 10.3389/fmed.2022.960689

**Published:** 2022-08-30

**Authors:** Lu Han, Hui Jia, Pingping Song, Xibin Liu, Zhendan Wang, Dujian Zhang

**Affiliations:** ^1^Department of Thoracic Surgery, Shandong Cancer Hospital and Institute, Shandong First Medical University and Shandong Academy of Medical Sciences, Jinan, China; ^2^Department of Respiratory Internal, Shandong Cancer Hospital and Institute, Shandong First Medical University and Shandong Academy of Medical Sciences, Jinan, China; ^3^Department of Surgery, Zaozhuang Tumour Hospital, Zaozhuang, China

**Keywords:** lung cancer, lymph node metastases (LN metastases), non-tumor-bearing lobe (NTBL) lymph nodes, non-tumor-bearing segment (NTBS) lymph nodes, outside tumor-bearing lobes and/or segments

## Abstract

**Objective:**

Hilar and lung lymph node metastases (N1) are defined as ipsilateral bronchial and intrapulmonary lymph nodes. However, the cleaning standards for ipsilateral bronchial lymph nodes in different lobes and segments within the same lobe in segmentectomy are not clearly defined.

**Materials and methods:**

Sixty-six patients undergoing pulmonary resection for the treatment of lung cancer were evaluated. Intraoperatively visible non-tumor-bearing lobe (NTBL) and post-operatively non-tumor-bearing segment (NTBS) lymph nodes were removed and analyzed. The associations between the NTBL LNs and clinicopathological characteristics were analyzed.

**Results:**

Non-tumor-bearing lobe LNs metastases were found in 8 (12.1%) of the 66 patients, NTBS LNs metastasis were not found (0/13). The presence of NTBL metastases was significantly associated with age (<60 years vs. ≥60 years, *P* = 0.037), differentiation (Grade 1 well differentiated vs. Grade 2 moderately differentiated vs. Grade 3 poorly differentiated, *P* = 0.012), CAT-scan-findings of Mediastinal and hilar lymph nodes metastasis (node-positive vs. node-negative, *P* = 0.022), pN stage (N0 vs. N1 vs. N2, *P* = 0.003) and p stage (I vs. II vs. III, *P* = 0.009). Multivariate logistic analysis showed that tumor differentiation (*P* = 0.048, HR 6.229; 95% CI 1.016–38.181) and pN (*P* = 0.024, HR 5.099; 95% CI 1.245–20.878) were statistically significant predictors.

**Conclusions:**

Lobar lymph node metastasis of NTBL occurs frequently in patients with NSCLC, but lymph node metastases in NTBS LNs are rare. Advanced age, poorly differentiated and N1 and N2 status of CAT-scan-findings were independent risk factors for the involvement of the NTBL lobar lymph nodes. Although lymph node metastases in NTBS are rare, further investigation of the need to dissect is required.

## Introduction

Lung cancer has the highest morbidity and mortality of all neoplastic diseases worldwide (1). Over two million new cases were diagnosed in 2018, making it the most common type of cancer. In China, a 40% increase in mortality is predicted between 2015 and 2030.

The standard surgical treatment for early stage non-small cell lung cancer (NSCLC) is lobectomy accompanied by systematic lymph node dissection (2, 3). The lymph node (LN) stage is critical for prognosis (4). In the National Comprehensive Cancer Network (NCCN) guidelines, systemic mediastinal LN (N2) clearance plays an important role in NSCLC surgical treatment, however, upgrading the staging after discovery of unexpected metastases in intrapulmonary LNs (N1), especially in non-primary tumor lobes and segments (NTBL/NTBS), has been reported (5–8). There are few reports on lobar lymph node metastases in NTBL/NTBS, for the following possible reasons: (1) The technique of intraoperative NTBL/NTBS LNs extraction is difficult with an increased likelihood of post-operative complications; (2) randomized trials have not demonstrated that LN resection provides more survival benefit than sampling (9, 10); and (3) As NTBL/NTBS LNs analysis is dependent on post-operative pathological examination rather than intraoperative assessment by the surgeons, incomplete resection may result (11). We hypothesized that patients undergoing lobectomy, anatomic segmentectomy, or wedge resection may have metastasized N1 LNs in NTBL/NTBS, affecting the post-operative pathological N staging. Our aim was to study the features of NTBL/NTBS LNs metastases and their correlation with clinicopathological features and clinical significance, to explore the necessity of NTBL/NTBS LN clearance.

## Materials and methods

### Patients

From March 2015 to March 2017, 66 consecutive patients undergoing pulmonary resection for lung cancer were evaluated. The study was approved by the Shandong Provincial Tumor Hospital Ethics Committee, and all patients signed prior informed consent. Inclusion criteria: (1) NSCLC patients requiring anatomical lung resection and mediastinal LNs dissection; (2) Lymph node dissection of at least 3 mediastinal lymph nodes, including subcarinal nodes, and simultaneous removal of intrapulmonary and hilar lymph nodes; and (3) For patients who have undergone NTBS LNs resection, the tumor size on CT was ≤3 cm. Exclusion criteria:(1) Patients with a previous history of other tumors or distant metastases; (2) Patients receiving preoperative chemotherapy, radiotherapy, or other anti-cancer treatment; (3) Patients undergoing sublobar resection who did not receive the systematic mediastinum and pulmonary LNs dissection; (4) Ipsilateral lung multiple lesions in patients; and (5) Small cell lung cancer patients. If any one of the above five exclusion criteria were met, the patient was not included in the group. The tumor size was divided into 4 groups (≤3 cm, 3∼≤5 cm, 6∼≤7 cm, and >7 cm) in accordance with the 8th Tumor Node Metastasis (TNM) classification for Lung Cancer (12).

### Lymph node evaluation

**FIGURE 1:**
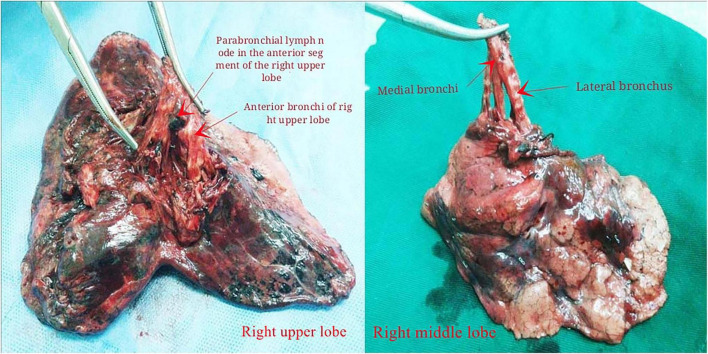
Lung bronchial tree specimens after lymph node dissection in right upper lobe and right middle lobe.

**FIGURE 2:**
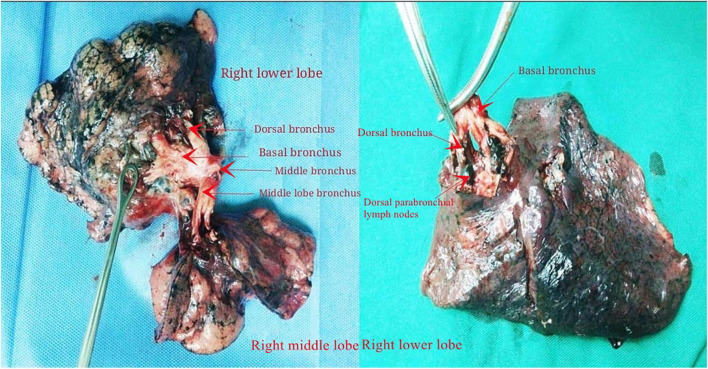
Lung bronchial tree specimens after intrapulmonary lymph node dissection in the right middle/lower lobe and right lower lobe.

Histopathologic analysis was performed by following the 2015 World Health Organization classification of tumors of the lung (13). The specific steps of lung LN clearance are as follows: first, we clear the hilar and mediastinal LNs 2–9 (at least 3 stations) mediastinal LNs cleaning is routinely implemented for cancer in the left and right lung cancer, respectively, paying special attention to the complete resection of LNs in Groups 10–11 of the lobes. The intraoperatively visible NTBL/NTBS LNs were removed and marked. The post-operative specimens were processed by experienced thoracic surgeons and pathologists, and intrapulmonary 12–14 LNs and NTBL/NTBS lymph nodes visible to the naked eye were identified and harvested (Figures 1, 2). The excised lobe’s bronchus was dissected along the proximal to the telecentric end to the segment bronchus; after determining the bronchial boundaries at all levels together with the inter-venous veins, visible intrapulmonary LNs were removed along the direction of the bronchial tree. The group, anatomical location, and numbers of removed LNs were recorded. All specimens were fixed in 10% formalin solution.

### Statistical analysis

Data were analyzed using SPSS (Version 22.0; IBM Corp., Armonk, NY, United States) and Excel. Continuous variables were expressed as mean ± standard deviation and differences analyzed using the *t*-test. The chi-square (χ^2^) test or Fisher’s exact test were used to investigate relationships between NTBL LNs and clinicopathological variables. *P* < 0.05 was considered statistically significant. Logistic regression models were built using a stepwise selection method. Multivariate analysis was used to identify independent predictors among the variables. The heat map is made by excel.

## Results

### Patient characteristics

The patient characteristics including age, sex, smoking history, serum carcinoembryonic antigen (CEA) levels, NSE levels, Cyfra21-1 levels, tumor differentiation cN2 stage, tumor size, lymph node metastasis, and staging are shown in Table 1.

**TABLE 1:** Demographics and clinical characteristics.

Parameter	*n*
**Age**	
<60	35
≥60	31
**Sex**	
Male	37
Female	29
**Smoking**	
Yes	34
No	32
**Histologic type**	
Squamous cell carcinoma	17
Adenocarcinoma	49
**CEA**	
Normal	39
Abnormal	27
**NSE**	
Normal	26
Abnormal	40
**Cyfra21-1**	
Normal	43
Abnormal	23
**Tumor size, cm**	
≤2	25
>2	41
**Differentiation**	
Grade 1, well-differentiated	9
Grade 2, moderately differentiated	37
Grade 3, poorly differentiated	20
**CAT-scan-findings of mediastinal and hilar lymph nodes metastasis**	
Node-positive	8
Node-negative	58
**pT stage**	
T1	21
T2	34
T3	11
**pN stage**	
N0	33
N1	13
N2	20
***p* stage**	
I	29
II	15
III	22

Among the 66 patients, VATS was performed on 45 patients and open thoracotomy was performed on 21 patients. NTBL metastases were found in 8 (12.1%) cases. The presence of NTBL metastasis was significantly associated with age (<60 years vs. ≥60 years, *P* = 0.037), differentiation (Grade 1 well differentiated vs. Grade 2 moderately differentiated vs. Grade 3 poorly differentiated, *P* = 0.012), CAT-scan-findings of Mediastinal and hilar lymph nodes (node-positive vs. node-negative, *P* = 0.022), pN stage (N0 vs. N1 vs. N2, *P* = 0.003), and p stage (I vs. II vs. III, *P* = 0.009) (Table 2). NTBL/NTBS metastases were not significantly associated with the patients’ sex, smoking history, CEA, NSE, Cyfra21-1, tumor size, or pT stage.

**TABLE 2:** Association between NTBL/NTBS and clinicopathological characteristics.

Parameter	Node-positive	Node-negative	*P*
Age			0.037
<60	7	28	
≥60	1	30	
Sex			0.695
Male	5	32	
Female	3	26	
Smoking			0.507
Yes	5	29	
No	3	29	
CEA			0.577
Normal	4	35	
Abnormal	4	23	
NSE			0.154
Normal	5	21	
Abnormal	3	37	
Cyfra21-1			0.867
Normal	5	38	
Abnormal	3	20	
Tumor size, cm			0.451
≤2	4	21	
>2	4	37	
Differentiation			0.012
Grade 1, well differentiated	0	9	
Grade 2, moderately differentiated	2	35	
Grade 3, poorly differentiated	6	14	
CAT-scan-findings of Mediastinal and hilar lymph nodes metastasis			0.022
Node-positive	4	7	
Node-negative	4	51	
pT stage			0.642
T1	3	20	
T2	3	30	
T3	2	8	
pN stage			0.003
N0	0	33	
N1	2	12	
N2	6	13	
p stage			0.009
I	0	29	
II	2	14	
III	6	15	

### Association between non-tumor-bearing lobe/non-tumor-bearing segment metastasis and tumor primary site

The frequencies of lymph node metastases did not differ significantly according to the primary site, left or right lung, or upper lobe vs. middle/lower lobe tumor (*P* = 0.619) (Table 3).

**TABLE 3:** Association between NTBL/NTBS metastasis and tumor primary site.

Location	Node-positive	Node-negative	*P*
Left lung upper lobe	1	10	0.619
Left lung lower lobe	4	14	
Right lung upper lobe	0	13	
Right lung middle lobe	1	3	
Right lung Lower lobe	2	18	
Total	8	58	

Univariate and multivariate logistic analyses were used to determine the impacts of the potential predictors of lymph node metastasis. Univariate analysis identified tumor differentiation (*P* = 0.013, HR 8.111; 95% CI 1.569–41.92), cN2 (*P* = 0.015, HR 7.286; 95% CI 1.478–35.915), pN (*P* = 0.008, HR 6.075; 95% CI 1.603–23.025), and p stage (*P* = 0.014, HR 5.485; 95% CI 1.42–21.184) as statistically significant predictors (Table 4). Further analysis with multivariate analysis showed that tumor differentiation (*P* = 0.048, HR 6.229; 95% CI 1.016–38.181) and pN (*P* = 0.024, HR 5.099; 95% CI 1.245–20.878) were statistically significant predictors.

**TABLE 4:** Univariate and multivariate logistic analyses the impacts of the potential predictors of lymph node metastasis.

	Univariate			Multivariate		
		
	Hazard ratio	95% CI	*P*-value	Hazard ratio	95% CI	*P*-value
Age (<60 years, ≥60 years)	0.133	0.015–1.153	0.067			
Sex (male, female)	0.738	0.161–3.383	0.696			
Smoking (yes, no)	0.6	0.131–2.746	0.51			
CEA (normal, abnormal)	1.522	0.346–6.701	0.579			
NSE (normal, abnormal)	0.341	0.074–1.57	0.167			
Cyfra21-1 (normal, abnormal)	1.14	0.247–5.267	0.867			
Tumor size (≤, 2, >, 2 cm)	0.913	0.39–2.136	0.833			
Differentiation (Grade 1, well; Grade 2, moderate; Grade 3, poor)	8.111	1.569–41.92	0.013	6.229	1.016–38.181	0.048
CAT-scan-findings of Mediastinal and hilar lymph nodes metastasis	7.286	1.478–35.915	0.015			
pT stage (T1, T2, T3)	1.573	0.526–4.703	0.418			
pN (N0, N1, N2)	6.075	1.603–23.025	0.008	5.099	1.245–20.878	0.024
*p* stage (I, II, III)	5.485	1.42–21.184	0.014			

## Discussion

The lymphatic drainage system of the lung was first described by Rouvière in 1932 (14, 15). In 1951, Cahan suggested that pneumonectomy together with hilar and mediastinal lymph node dissection should be a standard surgery for patients with lung cancer, which he termed “radical pneumonectomy” and, 9 years later, reported 48 cases of “radical lobectomy”, namely, lobectomy with regional lymph node dissection. In 1978, Naruke et al. (15) described a map numbering the lymph node stations from 1 to 9 for mediastinal (N2) stations and 10–14 for N1 stations. In 1996, after wide-ranging discussion, the term “Systematic Nodal Dissection” was accepted by the International Association for the Study of Lung Cancer (IASLC) (16). Subsequently, the accepted surgical treatment for NSCLC has been radical lobectomy, in other words, lobectomy combined with systematic lymph node dissection.

Lymph nodes may be assessed intraoperatively by one of five methods (17–19). The first is selected lymph node biopsy, which is usually used to prove N1 or N2 disease in cases, such as exploratory thoracotomy, where resection is not possible. Secondly, the Sampling-Sampling method involves the removal of one or more lymph nodes under the guidance of preoperative or intraoperative findings. In this case, the selection of lymph node stations is predetermined by the surgeon. Thirdly, systematic nodal dissection dissects and removes all the mediastinal tissue containing the lymph nodes. Fourthly, lobe-specific systematic node dissection involves the excision of the mediastinal tissue and lymph nodes, depending on the lobar position of the primary tumor, and, fifthly, extended lymph node dissection in which the bilateral mediastinal and cervical lymph nodes are removed.

There are various guidelines for the extent of lymph node resection. The NCCN guidelines stipulate sampling a minimum of three N2 stations or total mediastinal dissection (20). The Commission on Cancer (CoC) recommends the removal and analysis of at least 10 lymph nodes (21) while the European Society of Thoracic Surgeons recommends mediastinal dissection of all lymph nodes (22). According to the International Association for the Study of Lung Cancer (IASLC) Staging Committee recommendations, there must be no extracapsular extension of the tumor and the highest mediastinal node must test negative after removal. (23).

Despite the differences in current intraoperative lymph node assessments, systematic lymph node dissection is the gold standard. Using this, some scholars have found unexpected metastases in intrapulmonary LNs (N1) especially in NTBL/NTBS LNs (24). The metastatic characteristics and impacts on the patient prognosis of NTBL/NTBS LNs are unknown.

**FIGURE 3:**
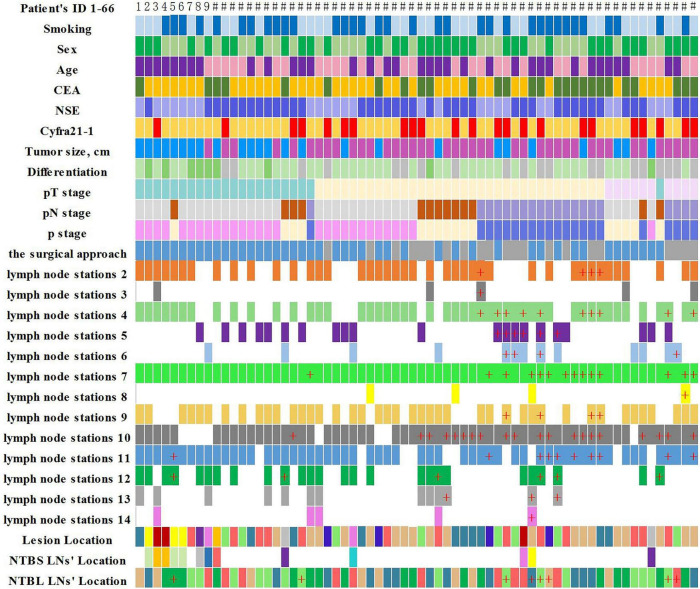
Clinicopathological features and lymph node metastasis of the 66 lung cancer patients (“+” represents positive lymph node metastasis).

**FIGURE 4:**
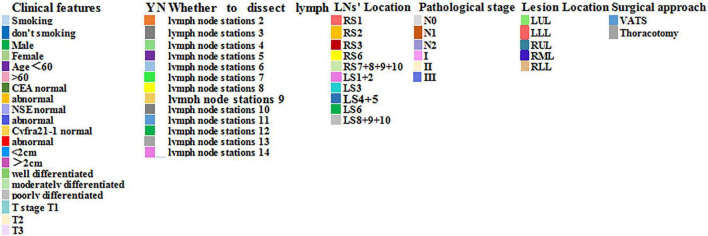
Meaning of different colors.

In our study, NSCLC patients requiring anatomical lung resection and mediastinal LNs dissection. Lymph node dissection of at least 3 mediastinal lymph nodes, including subcarinal nodes, and simultaneous removal of intrapulmonary and hilar lymph nodes. Clinicopathological features and lymph node metastasis of disease in 66 lung cancer patients are shown in Figures 3, 4.

NTBL LNs metastases were found in 8 (12.1%) of the 66 patients. Yamanaka et al. (9) and Liu et al. (24) reported that the incidence of lobar lymph node metastases in NTBL LNs were 10.13 and 12.6%. Liu et al. inferred that these extended lymph node metastases were mainly secondary to lymphatic congestion (24). It is worth noting that one patient in our study was found to have an isolated NTBL LNs metastasis, and his pathological stage was upgraded from I to II b, which has an important impact on his treatment strategy. This patient’s preoperative CT showed a solid component-based nodule, and the post-operative pathology showed poorly differentiated squamous cell carcinoma of the lung. Robinson et al. (25). reported the frequent occurrence of occult nodal disease in peripheral N1 stations (11–13) in NSCLC patients with peripheral tumors less than 2 cm diameter and no apparent metastatic spread (T1a-bN0M0). Combined with our research, this suggests that lymph node metastasis in clinical T1a-bN0M0 patients can occur not only in the primary tumor N1, N2 station but also in NTBL LNs. It is well known that the number of positive LNs is an independent prognostic factor of survival in patients with N1 NSCLC (26). NTBL can increase the detection rate of positive lymph nodes, which not only can reduce local recurrence rate but also predict patient prognosis. Yamanaka et al. (9). suggested dissection of all lobar lymph nodes in the remaining NTBL LNs in patients with lobar-hilar or multistation mediastinal lymph node metastases, particularly those on the right side.

No patients (0/13) with NTBS LNs metastasis were found. Sakairi et al. (27). and Lin (28) reported incidences of lymph node metastases in NTBS LNs of 1.6 and 1.2%, respectively. In clinical stage IA, the incidence of peripheral lung cancer lymph node metastases in NTBS LNs was found to be 1.0% (28). These research data are consistent with our findings regardless of the differences in staging. Metastasized lymph nodes occur infrequently in NTBS LNs and the necessity of the cleaning of NTBS LNs lymph nodes during surgery requires further research.

Interestingly, our statistical analyses showed that NTBL LNs metastases are associated with CAT-scan-findings of Mediastinal and hilar lymph nodes metastasis and not associated with location. NTBL lobar lymph node metastasis was most often observed among patients with larger tumor size, N1/N2 nodal involvement, with lymph vascular invasion (LVI), and visceral pleural invasion (VPI) (24). Because of this, we consider NTBL LNs resection to be of vital importance for these patients in terms of accurate staging and reduction of the post-operative local recurrence rate. In addition, NTBL LNs showed a significant correlation with tumor tissue differentiation (*P* = 0.013), which is of great significance for guiding the patients’ prognosis and post-operative management.

Our research shows that lobar lymph node metastasis of NTBL occurs frequently in patients with NSCLC, but lymph node metastases in NTBS LNs are rare. Advanced age, poorly differentiated and N1 and N2 status of CAT-scan-findings were independent risk factors for the involvement of the NTBL lobar lymph nodes. ^18^F-FDG PET/CT provides high sensitivity and accuracy, it has overtaken CT as the imaging source of choice for preoperative staging (29). In our study, PET-CT is performed in 8 of 66 patients, but unfortunately, none of these 8 patients have NTBS/NTBL lymph nodes metastasis in post-operative pathology, otherwise, we can further analyze the guiding significance of PET-CT in NTBL/NTBS lymphadenectomy. This is very important. Further investigation into the necessity of NTBL/NTBS dissection is required. We will continue to follow up patients to explore the clinical significance of NTBL/NTBS LNs.

## Data availability statement

The original contributions presented in this study are included in the article/supplementary material, further inquiries can be directed to the corresponding author.

## Ethics statement

This study was approved by the Shandong Provincial Tumor Hospital Ethics Committee. The patients/participants provided their written informed consent to participate in this study. Written informed consent was obtained from the individual(s) for the publication of any potentially identifiable images or data included in this article.

## Author contributions

HJ was in charge of project design. LH collected, analyzes data, and wrote the manuscripts. PS and XL performed the operation and analyzes the data. ZW and DZ used software to analyze data. All authors contributed to the article and approved the submitted version.
